# Continuous Implant Load Monitoring to Assess Bone Healing Status—Evidence from Animal Testing

**DOI:** 10.3390/medicina58070858

**Published:** 2022-06-27

**Authors:** Markus Windolf, Viktor Varjas, Dominic Gehweiler, Ronald Schwyn, Daniel Arens, Caroline Constant, Stephan Zeiter, Robert Geoff Richards, Manuela Ernst

**Affiliations:** AO Research Institute Davos, 7270 Davos, Switzerland; viktor.varjas@aofoundation.org (V.V.); dominic.gehweiler@aofoundation.org (D.G.); ronald.schwyn@aofoundation.org (R.S.); daniel.arens@aofoundation.org (D.A.); caroline.constant@aofoundation.org (C.C.); stephan.zeiter@aofoundation.org (S.Z.); geoff.richards@aofoundation.org (R.G.R.); manuela.ernst@aofoundation.org (M.E.)

**Keywords:** implant load monitoring, fracture monitoring, implantable sensor, nonunion, bone plate, bone fracture

## Abstract

*Background and Objectives*: Fracture healing is currently assessed through qualitative evaluation of radiographic images, which is highly subjective in nature. Radiographs can only provide snapshots in time, which are limited due to logistics and radiation exposure. We recently proposed assessing the bone healing status through continuous monitoring of the implant load, utilizing an implanted sensor system, the Fracture Monitor. The device telemetrically transmits statistically derived implant parameters via the patient’s mobile phone to assist physicians in diagnostics and treatment decision-making. This preclinical study aims to systematically investigate the device safety and performance in an animal setting. *Materials and Methods*: Mid-shaft tibial osteotomies of different sizes (0.6–30 mm) were created in eleven Swiss mountain sheep. The bones were stabilized with either a conventional Titanium or stainless-steel locking plate equipped with a Fracture Monitor. Data were continuously collected over the device’s lifetime. Conventional radiographs and clinical CT scans were taken longitudinally over the study period. The radiographs were systematically scored and CTs were evaluated for normalized bone volume in the defect. The animals were euthanized after 9 months. The sensor output was correlated with the radiologic parameters. Tissue samples from the device location were histologically examined. *Results*: The sensors functioned autonomously for 6.5–8.4 months until energy depletion. No macroscopic or microscopic adverse effects from device implantation were observed. The relative implant loads at 4 and 8 weeks post-operation correlated significantly with the radiographic scores and with the normalized bone volume metric. *Conclusions*: Continuous implant load monitoring appears as a relevant approach to support and objectify fracture healing assessments and carries a strong potential to enable patient-tailored rehabilitation in the future.

## 1. Introduction

Over the past decades, internal fixation of bone fractures has evolved into a well-managed procedure and has transformed the incident of a skeletal fracture from a disabling and sometimes life-threatening event into an, for most of the cases, inconvenient but manageable episode. Still, significant rates of severe complications related to internal fixations persist, including hardware failures and non- and delayed-unions of 5–10% [1]. The risk factors for healing disturbances are manyfold, including open fractures, impaired blood supply, infections, diabetes, smoking, alcohol abuse and inadequate fixation stability, to name the most important [2,3]. Emphasis for improvements to prevent complications has been put mainly on surgical procedures and implant development. This is surprising, since after-treatment builds an essential pillar of the patient journey, potentially determining its success. Advances in aftercare and rehabilitation lack behind and follow decades-old protocols based on generalized beliefs and conventions. Individualized and evidence-based rehabilitation, particularly in the early healing phase, (1) may prevent complications in the first place through patient-tailored physiotherapy and mobilization or (2) may enable early diagnosis of complications for taking timely counteractions. These include adapting physiotherapy or applying therapeutic noninvasive (e.g., shockwave or ultrasound therapies) or invasive treatments. The timely detection of healing disturbances is regarded as essential puzzle piece for fast recovery. The FDA defines a nonunion as a fracture that remains ununited after 9 months [2]. This convention is, however, under debate within the medical community towards shortening the period to 6 months [4]. Regardless, the approach creates unproductive waiting time for the patient and obviously forms a social-economic burden [1].

Novel assessment and feedback modalities for improving rehabilitation are still rare. Approaches include measuring insoles or body-worn accelerometer-based sensors, but state-of-the-art fracture follow-up is still based on physical examination and radiographic screening. X-ray imaging offers advantages through the visualization of fracture dislocation, heterogenic ossification, or implant failure localization—parameters that sensor measurements cannot provide. On the other hand, the shortcomings are eminent. Radiographs are highly subjective to interpretation and are taken at distinct timepoints, thus capturing only a few snapshots of the healing over time, and bind the patient to hospital visits when home care would be the preferable mode. Self-speaking, radiographs expose individuals to radiation, which should be reduced to a minimum.

The temporary stabilizing function of trauma implants provides another option for assessing bone healing progression. A decline in implant load (or respective implant deflection) under physiological loading may be interpreted as an indirect measure for fracture healing when the bone, from an initially broken state, gradually takes over and unloads the implant. The principle has been researched over the past decades in preclinical and clinical settings using strain gauge-instrumented implants. Pioneered by Burny [5] and Jorgensen [6] on external fixation, the concept was taken on by various other groups, e.g., by Claes [7], and applied internally on an instrumented plate by Seide et al. [8] and Kienast et al. [9]. So far, these were mainly passive devices requiring external power induction. Even though the approach provides a clear advantage over radiographic imaging, it still remains a noncontinuous “snapshot” procedure. We believe that continuous measurement of implant loading is required to exploit the full potential to improve and individualize rehabilitation. We therefore proposed a concept for continuous monitoring of the implant parameters [10,11], developed it into a sensor system for use with external fixators and collected a first promising set of clinical data on infected segment transport patients [12]. The Fracture Monitor is currently developed into a human implantable version for use with conventional angular stable locking plates under consideration of the European regulatory framework for class III medical devices. The aim of this study was to systematically investigate the device safety and performance in a large animal experiment to provide evidence for interpretability of the sensor output with regards to the bone healing status on the path to clinical introduction.

## 2. Materials and Methods

### 2.1. Fracture Monitor System

The Fracture Monitor was developed in the frame of our ISO13485 certified quality management system for medical devices. It consists of an implantable datalogger, a smartphone app for wireless communication with the datalogger via Bluetooth Low-Energy and a cloud server for centralized data collection.

**Figure 1:**
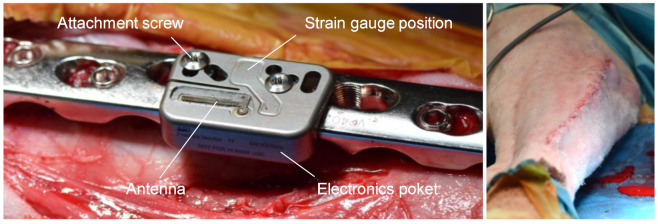
(**Left**): Implantable datalogger attached to a conventional stainless-steel locking plate with attachment screws and inserts (not visible) during surgery. Strain gauges are located in between the attachment screws to pick up implant load. The electronics pocket accommodates the electrical components and battery. An epoxy-potted antenna transmits data using the Bluetooth Low-Energy protocol. (**Right**): Sheep leg after wound closure above the implant.

The implantable datalogger incorporates a set of resistive foil strain gauges positioned in between two screw holes for bone plate attachment. The strain gauges are configured to pick up bending and tension at the bone plate surface, considered as predominant loading modes. The datalogger is attached by two sets of custom-made 5-mm conical inserts and attachment screws. The sensor is incorporated in a Titanium grade 5 alloy housing hermetically sealed by means of laser welding. Biocompatibility of the data-logger and fixation elements is verified according to the ISO10993 test series. The sensor constitutes an L-shape, where bulkier electronics are enclosed in a pocket beside the bone plate, leaving only a thin section of <2.5-mm prominence above the plate. A pattern of holes allows fitting the sensor to a variety of commercially available locking plates (Figure 1). A strain-gauge Wheatstone half-bridge is amplified, sampled at 10 Hz and processed by a low-power microprocessor. A peak detection algorithm identifies loading events in real-time in the measured signal, defined as the minimum value, followed by the maximum above a predefined amplitude threshold and a subsequent relative drop of the signal below a predefined level. Only statistical parameters derived from this raw data are written to the internal nonvolatile memory to keep energy consumption for data storage and transmission at a minimum. These parameters remain permanently stored over the lifetime of the device to avoid data loss. The statistical parameters include, among others: (1) average buffer amplitude, defined as the average of the 50 largest loading amplitudes (minimum-to-maximum difference) over 24 h; (2) average minimum load, defined as the average minimum value of all loading events over 24 h; (3) peak load, defined as the highest observed loading amplitude over 24 h; and (4) amplitude histogram: event counts at different amplitudes over 24 h. An integrated real-time clock ensures accurate timing of the system. To track patient activity and to further reduce energy consumption an onboard low-power accelerometer is used to control wake up and sleep periods of the microprocessor according to movement of the patient’s leg. Percentage of active time over the day is logged and transmitted as well. The embedded software of the datalogger was developed and validated according to the IEC62304 medical device software lifecycle requirements. The epoxy potted antenna was matched to the dielectric environment of the body and is connected to a Bluetooth Low-Energy transceiver. The radio module was tested and certified for electromagnetic compatibility according to the requirements of IEC60601-1 and -2 and of the radio equipment directive (RED). The datalogger is actively powered by a sealed Li-Ion battery with a single charge over the device lifetime and, hence, allows autonomous and continuous (24 h) data monitoring. At a critical battery charge, the device switches to an energy-saving mode, where monitoring is performed only one day per week and otherwise suspended. A magnet incorporated in the blister package disables a reed switch to allow power up through the hermetic enclosure at the time of implantation. The device complies with the general requirements for active implantable medical devices (ISO14708-1).

The smartphone application is compatible with Android consumer devices and attempts a connection once a day to download most recent statistical data from the implanted datalogger. The time of download is independent from the measurement and can be freely chosen. A foreground service on the mobile phone automizes this process to allow fully autonomous data transfer without required patient interaction. For security reasons, the smartphone app acts as a data gateway only. Several data security and misuse protection measures were put in place, incl. password-protected access, dynamic data encryption, unidirectional communication, temporary data storage, singular and proprietary Bluetooth bonding, to name a few. The app was tested for usability aspects for later use in a clinical setting.

The smartphone application automatically forwards the collected data to a dedicated cloud environment where patient data can be allocated to physician accounts for data visualization. Mean buffer amplitude is post-processed by applying a 7-day moving average filter (trailing) and normalizing the unitless, uncalibrated sensor data to their maximum over the measurement period, referred to as “relative implant load” (RIL).

### 2.2. Animal Experiment

The Fracture Monitor system was used in an animal experiment in eleven skeletally mature female Swiss White Alpine sheep (age: 3.6 ± 0.8 years; weight: 69.5 ± 6.2 kg (mean ± SD)) to assess in vivo performance and safety of the system throughout the device lifetime. An ovine tibia osteotomy model with plate osteosynthesis and osteotomy widths from 0.6 mm to 30 mm was employed to which the animals were randomly allocated. The operational side (left or right limb) was randomized as well. Together with the use of either a 10-hole 4.5-mm Titanium broad LCP Plate (REF: 426.601, DePuySynthes Inc., Raynham, MA, USA) or a 10-hole 5.5-mm stainless-steel veterinary broad LCP plate (REF: VP4071.10, DePuySynthes Inc., Raynham, MA, USA), a range of different healing conditions in terms of fixation stiffness and defect size was created. The study was performed in an AAALAC International accredited facility in compliance with the Swiss animal welfare regulations and was approved by the local responsible ethics committee (Canton of Grisons, Switzerland, approval: TVB GR 2019/11). At least two weeks prior to study start, the sheep were housed in groups in the facility for acclimatization to the study conditions.

#### 2.2.1. Device Preparation

Prior to surgery, the dataloggers were mounted to the respective locking compression plates for calibration and function testing. The plates were attached to Canevasit rods (25 mm diameter) as bone substitute in the same configuration as used later in vivo. The construct was axially compressed on a material testing machine from 0 N to 1000 N. Load was introduced to the center of the rods via metal spheres to mimic a free center of rotation at the joints. Individual construct sensitivity (sensor with plate configuration) in terms of raw signal units (least significant bits, LSB) per kg was calculated. Devices were cleaned, packaged and sterilized with Ethylene-oxide to prepare implantation.

#### 2.2.2. Surgical Procedure

After sedation with Detomidine (0.04 mg/kg IM (intermuscular)) and induction using Midazolam (0.2 mg/kg IV (intravenous)) and Ketamine (4 mg/kg IV), the surgery was performed under general anesthesia using gas anesthetic (1.5–2.0% Sevoflurane in approximately 75% oxygen) with the animal’s vital parameters monitored continuously during the procedure. Preemptive analgesia consisted of Lidocaine (2 mg/kg) and Buprenorphine (0.005 mg/kg) epidurally, as well as Carprofen (4 mg/kg IV). The sheep was placed in lateral recumbency, and the tibia was aseptically prepared. A skin incision was made on the medial aspect of the tibia followed by blunt subcutaneous dissection to expose the bone. The LCP plate (Titanium or steel) was distally pre-bent, placed on the medial tibia into a best-fit position and fixed with 5.0-mm locking screws (three screws proximally, four screws distally, REF: 423.3xx/213.3xx, DePuySynthes Inc.). The soft tissues around the tibia at the expected osteotomy/ostectomy site were circumferentially detached from the bone leaving the periosteum intact. After plate fixation, a transverse osteotomy/ostectomy of either 0.6, 2, 4, 6, 10 or 30 mm was created using an oscillating saw and a custom-made jig centered on the plate between holes 5 and 6. After placing the custom-made conical inserts into the empty plate holes 4 and 5 from proximal, the datalogger was attached with custom screws under torque control (1.5 Nm, Figure 1) before closing the surgical wound in 3 layers with absorbable suture material. Refer to Table 1 for assignment of plate materials, operational side and osteotomy/ostectomy sizes. All animals received Carprofen (4 mg/kg SC q 24 h for 5 days), Buprenorphine (0.05 mg/kg IM q 8 h for 3 days) and Fentanyl (2 µg/kg/h for 72 h, patches) as postoperative analgesia.

#### 2.2.3. Post-Operative and Post-Mortem Protocol

**Figure 2:**
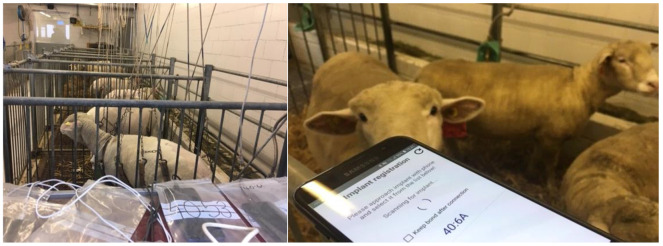
(**Left**): animals in single boxes under sling support during the first 5 weeks post-operation. A smartphone (in plastic bag) per animal was kept in the same room for automatic data transfer. (**Right**): connection attempt to a datalogger for data streaming.

Animal welfare assessment was performed and recorded using a score sheet twice daily for the first 3 days postoperatively, followed by daily evaluation for 4 additional days and then once weekly. The sheep’s weight was also monitored weekly. The animals were allowed immediate full weight-bearing after surgery but were kept in a loose harness to avoid excessive loads on the operated leg for the first 5 weeks (animal with 30 mm ostectomy for the entire study). Afterwards, they were housed in small groups. On first day after surgery, the datalogger of each animal was registered to a dedicated consumer smartphone (Android operating system, various brands and models) via the Fracture Monitor app. The smartphones were kept on a shelf in the same room. Thereon, dataloggers transmitted the recorded parameters daily to the smartphones (Figure 2). Time to a relative implant load drop of ≥50% was evaluated for each animal, if applicable. After 4 weeks, live sensor signal streaming was performed with the animals walking in the hallway. An example recording is given as Supplementary Material Video S1.

Plain anteroposterior (AP), mediolateral (ML) and oblique radiographs were taken post-operatively, then weekly for the first three months and monthly thereafter. Radiographs of eight animals were retrospectively scored according to the modified RUST score [13] (mRUST, three visible cortices, Table 2) by two independent assessors with orthopedic training. The three remaining animals could not be scored because oblique radiographs were not taken. Spearman’s correlation coefficient R (nonparametric) between the RIL and mRUST scores was calculated for both assessors.

Clinical CT scans (Revolution EVO, GE Medical Systems (Schweiz) AG, Glattbrugg, Switzerland) with 0.625 mm slice thickness, 120 kVp, and bone reconstruction kernel were taken post-operatively, then monthly for the first four months and directly after euthanasia. Scans were acquired with a bone mineral density calibration phantom (QRM-BDC/6). Bone volume was calculated for the half-tube opposite the bone plate to minimize metal artifact influence on the evaluation. Bone volume was normalized to the gap size for comparability between animals. Pearson’s correlation coefficient R between RIL and normalized bone volume was calculated for 4 and 8 weeks post-operation, when RIL drop was still incomplete to enable correlation analysis.

The sheep were euthanized 9 months after surgery by means on an intravenous overdose of Barbiturate (7.5 g IV). Both tibiae were harvested for postmortem evaluations. During dissection, a macroscopic examination for soft tissue changes was performed. Tissue samples for histological analysis were collected from the direct vicinity of the datalogger and from a distant location (above plate hole 9, distal end of the plate) as an internal control. The soft tissue samples were first stored in 4% buffered formaldehyde and then processed further for paraffin embedding. Using a paraffin microtome, sections of 5 µm thickness were made. For each sample one section was stained with Hematoxylin and Eosin and another with Prussian Blue reaction.

Intact and operated tibiae were cut proximally and distally to a remaining shaft section of 10.5 cm after soft tissue removal and were embedded in Polymethylmethacrylate. Samples were biomechanically tested to failure in quasistatic external rotation through cardan joints on a material testing machine (858 Mini Bionix, MTS Systems Corp., Eden Prairie, MN, USA). Torque to failure and torsional stiffness were evaluated from the angle-toque curves and normalized to the unoperated tibiae for relative comparison.

## 3. Results

Surgical procedure and Fracture Monitor implants were tolerated well. Animals recovered uneventfully. Except for the animal with the 30 mm ostectomy, all animals healed through periosteal callus formation and achieved bony union during the study period. This was confirmed by mechanical testing of the fractured tibiae reaching a mean torque to failure of 99 ± 30% and a mean torsional stiffness of 107 ± 38% of the intact contralateral bone. The tibia of the animal with the 30-mm defect could not be mechanically tested due to insufficient stability after plate removal. In one animal, the custom-made conical inserts became loose during the first week post-surgery. This was corrected in a secondary minimal invasive revision three weeks post-operation by retightening the inserts and reattaching the implantable datalogger.

**Figure 3:**
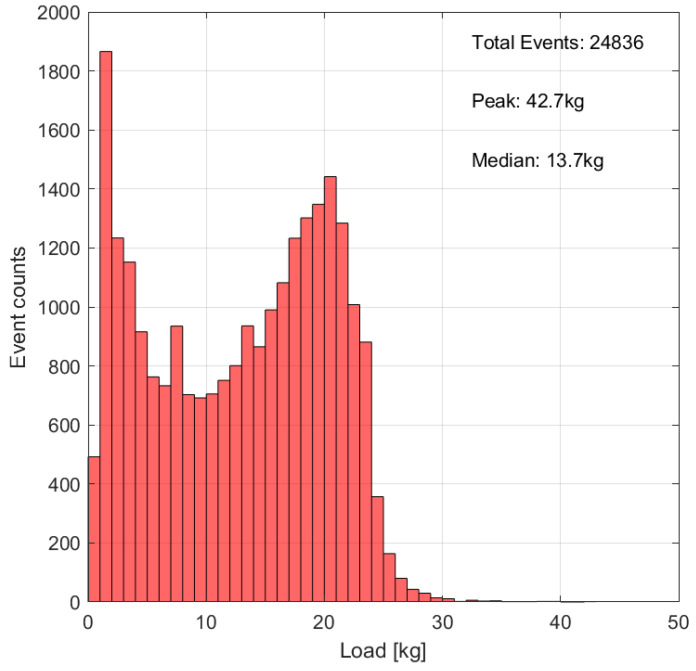
Exemplary loading event histogram over the first 7 days of measurement with calibrated load-bins. Bin width 1 kg, animal 519,042.

All dataloggers kept functioning flawlessly and autonomously throughout the study until complete battery discharge. All data were successfully synchronized to the connected smartphones with a median connection time of <4 s per day. The average monitoring period until battery depletion was 230 days (range 197–255 days), divided into an active monitoring phase of, in average, 124 days, followed by a power–safe phase of 106 days. Animal movement kept the sensors activated for, on average, 6.5% active time over 24 h. Active time was consistently highest on the first day after surgery with up to 53%. On average, 1294 loading events per day were detected (range 872–1915). An exemplary histogram of loading event intensity distribution over the first 7 days of measurement is given in Figure 3. Device sensitivity, used for calibrating the raw sensor signal to load in kg ranged between 14.2 LSB/kg (steel construct) and 36.2 LSB/kg (Titanium construct). Details on sensor parameters are given in Table 1.

**Figure 4:**
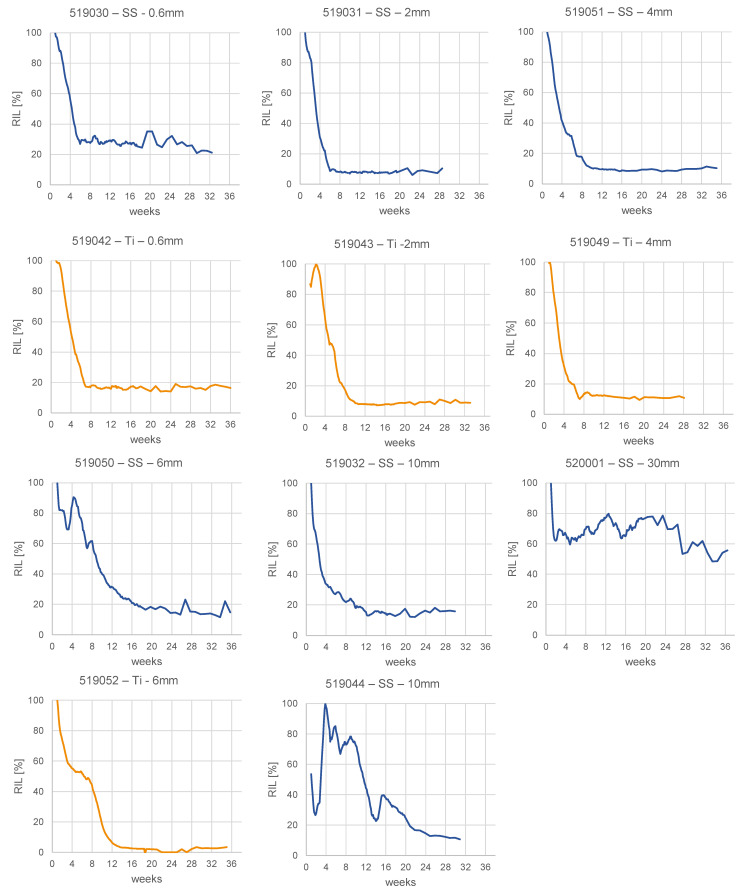
Relative implant load (RIL) curves for all animals in the study. Constructs with Titanium (Ti) plates in orange; stainless steel (SS) constructs in blue. Osteotomy gap sizes given in mm. Animal 519,044 was reoperated 3 weeks post-operation.

Within the monitoring period relative implant load dropped at least to 21% of the maximum level in all animals, except the animal with the largest (30 mm) ostectomy width, which reached 56% RIL at the end of the study (Figure 4). The median time to a relative implant load drop of 50% was 31 days.

**Figure 5:**
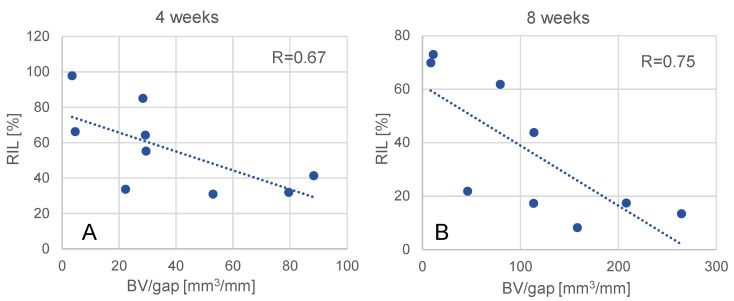
Correlation between relative implant load (RIL) and normalized bone volume (BV/gap) at 4 weeks (**A**) and 8 weeks (**B**).

Due to the resolution limit of the CT scanner (slice thickness 0.625 mm) normalized bone volume could not be evaluated for the 0.6 mm gap animals. For the remaining nine datapoints, relative implant load correlated significantly with the normalized bone volume at 4 weeks (R = 0.67, *p* = 0.05) and 8 weeks (R = 0.75, *p* = 0.019) post-op (Figure 5).

**Figure 6:**
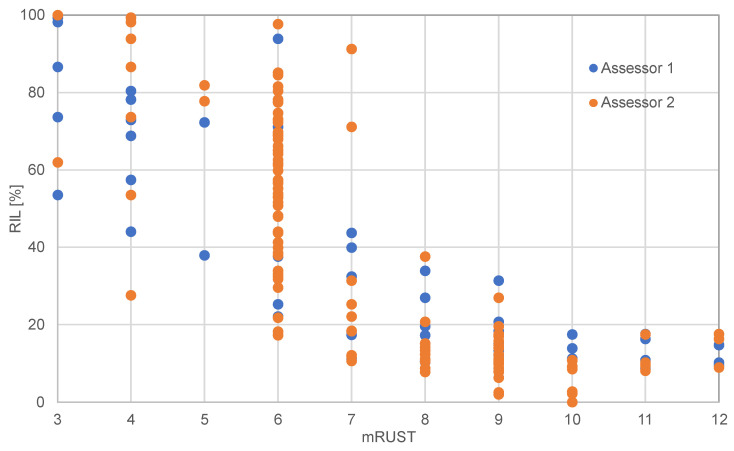
Correlation between radiographic scoring (mRUST) and relative implant load (RIL) for two assessors. Eight animals included (519,042–520,001).

**Figure 7:**
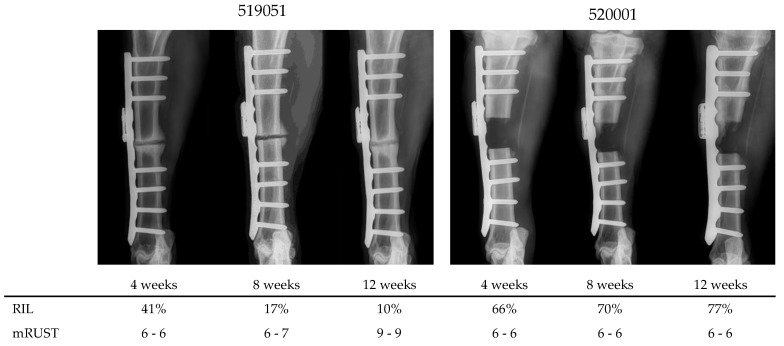
Exemplary sequences of radiographs in AP view at 4, 8 and 12 weeks post-operation of two animals and corresponding relative implant load (RIL) and mRUST scores (assessor 1–assessor 2).

The correlation coefficients for mRUST and RIL were R = 0.855 and R = 0.845 for the two assessors respectively (Figure 6). Both correlations were significant (both *p* < 0.001). At the time when relative implant load dropped to 50%, the corresponding mRUST score was 6 (callus present but not bridging, fracture line visible in all three cortices) for all animals. Exemplary X-ray sequences are shown in Figure 7.

**Figure 8:**
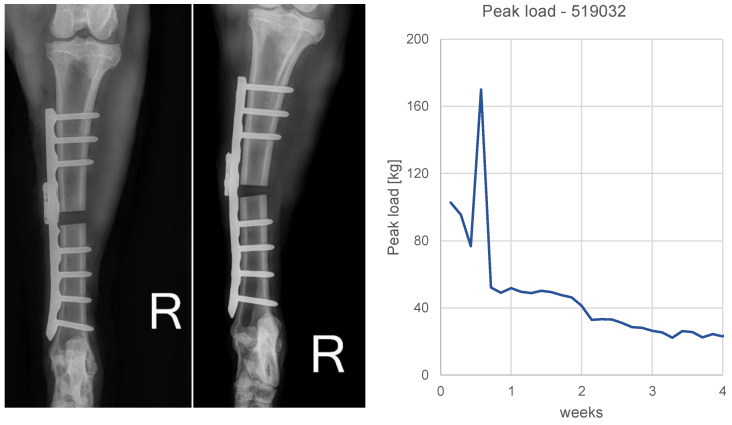
Plate bending in animal 519,032. (**Left**): Post-op and 1-week AP radiographs. Plastic deformation of the plate after 1 week becomes apparent. (**Right**): calibrated daily peak load sensor data of the first 4 weeks. A spike (170 kg) at day 4 is visible.

In animal 519,032 a single spike load event (170 kg) was seen in the data at day 4 post-operation. The subsequent 1-week control image exhibited moderate plate bending. The animal was kept in the study (Figure 8).

Macroscopic and histological analyses did not reveal critical observations regarding long-term biocompatibility or occurrence of corrosion or wear particles related to the Fracture Monitor implants. There were no differences found on the tissue samples taken from the datalogger surrounding and from the control location.

## 4. Discussion

The Fracture Monitor system intends to assess bone healing status through continuous measurement of implant load. This animal study aimed at investigating safety and performance of the device in a preclinical setting. We found significant correlations between the primary output metric of the system, relative implant load (RIL) and radiologic parameters derived from plain radiographs and CT. This observation is regarded as proof of the measurement principle. To simulate diverse healing, the size of the experimental osteotomy gap was varied between 0.6 and 30 mm. Even though not considered a clinically realistic nonunion model, the approach is deemed suitable for the purpose of creating a range of mechanical healing conditions, including the situation of a non-healing fracture with the 30 mm defect. By additionally utilizing two plate materials with approx. 100% elasticity difference, Titanium and stainless steel, a mechanical healing scenario with maximum variance was created to fulfill the purpose of the study. As indicated by mechanical testing of the specimens at the end of the experiment, all bones fully consolidated despite the animal with the 30-mm defect, which did not heal.

Core element of the Fracture Monitor system is an implantable datalogger, which is attached to conventional bone plates and, hence, does not interfere with established clinical practices. The surgeon may continue using standard operation techniques and implants and may decide upon additionally implanting a Fracture Monitor on an individual case basis. The device is intended to be implanted and explanted together with the fixation hardware, thus avoiding additional surgical procedures. For the case that the orthopedic implant is left in situ, the sensor was designed following manufacturing strategies commonly used for permanently implanted active medical devices, such as pacemakers. This includes use of a hermetic metallic package providing a long-term non-degrading barrier against ingress and egress of substances. Communication is enabled through a potted antenna with hermetic electrical feedthrough comparable to a pacemaker header. We decided to use a consumer wireless communication protocol, Bluetooth Low-Energy, despite obvious challenges. Firstly, the Bluetooth frequency band at 2.4 Ghz is subject to considerable damping and transmission range loss when transmitted through body tissues. After antenna matching and minimizing return loss, the achievable transmission distance through tissues was approx. 6 cm below the skin. This, however, is from our analysis sufficient for the vast majority of anatomical locations and anticipated soft-tissue coverages. Here, with the sensor implanted in the sheep limb, the data transmission functioned reliably with the receiver smartphones located apart from the animal in the same room. Secondly, Bluetooth communication remains to a great extent a black box technology. Especially on the smartphone side communication performance and reliability can vary significantly with device brands, models and automatic software updates. With developing a proprietary pairing protocol, we found an acceptable compromise for reliability, data security and performance. The outweighing advantage of using this standard is seen in the seamless embedding of medical technologies into the patient’s life. Use of the personal smartphone is key and allows real-time and remote monitoring of health parameters, without the need for frequent hospital visits. This subjectively renders the episode of the injury and treatment as short as possible and, at the same time, enables the physician to closely follow-up the patient. Continuous data collection is regarded fundamental for remote monitoring and is the key-characteristic of the proposed technology. It allows not only timely diagnostics and therapeutic decision making, but also enables tailoring rehabilitation protocols to the individual patient need. Activity profiles, such as loading event histograms, as exemplified in Figure 3, may provide useful information to adapt after-care in the future. Continuous data collection requires an active power supply, in our case an implanted battery. Power management was laid out to cover the normal episode of fracture healing. In this sheep experiment device lifetime was between 6.5 and 8.4 months. Given the purpose of the system, preventing long-lasting healing complications, this is considered sufficient. However, device lifetime is a function of patient activity, which might be different in humans.

Continuous implant load monitoring has also an important technical justification. Other solutions are passively powered and, hence, allow only snapshot measurements at distinct timepoints. When conclusions on the healing state are targeted, the influence of varying patient loading needs to be taken into account. Implant load measurement with short data snippets can be biased by physiological loading at the time. Reference measurements such as ground reaction forces are required, which complicate the procedure for homecare and remote monitoring. Our system remains simple, with a single sensor and automatic data collection without required patient interaction. The results suggest that with continuous measurement natural variances of patient loading can be averaged out to a certain extent to avoid obstruction of the load-shifting effect to the healing bone. The measured RIL curves consistently show a distinct and robust response at the early healing phase (median drop to 50% RIL within 31 days) even though animal activity was left uncontrolled. Animals could weight-bear at discretion. Also, the nonhealing animal (520001, 30-mm defect) showed an accentuated drop in the direct post-operative phase (Figure 4), which occurred too early to be attributed to stiffening effects in the fracture. It is likely due to a reduced loading magnitude after extensive activity under pain suppression. Activity and physiological loading changes of the patient must be considered when interpreting the data for bone healing status.

Relating implant strain to bone healing has been followed-up in a research context over decades. In the 1960/70s strain gauge data have first been reported on external fixator patients due to the good accessibility of the fixation [5,6,14,15] and followed-up in the coming decades [7,16,17,18,19,20,21]. In 1984, Burny et al. summarized the outcomes of >500 patient datasets and defined healing categories (fast, normal, slow, pseudarthrosis) based on the decline of the curves [22]. Claes at al. reported on another 100 external fixation patients and concluded that the method can allow for early detection of patients at risk for healing disturbances [16]. Schmickal et al. [20] stated later from the same dataset that a drop of the loading curve below 40% of its maximum determined the faith of healing. When falling below this margin all fractures robustly consolidated. This stresses two points: (1) A relative representation of the implant load progression appears superior to absolute force or strain measurements. This simplifies the procedure, because calibration is not essentially required. We consequently normalized the sensor data to relative implant load; (2) Capturing the global maximum of the implant load curve is important speaking in favor of continuous implant load measurement. Furthermore, Claes et al. [16] found that the mechanical measurement responded earlier than healing became apparent on radiographs. An observation, which is confirmed by the comparison between RIL and radio-logic scoring. Even in this highly controlled setting, particularly at early stages mRUST assessment appeared diffuse and subjective, while RIL drops distinctly. This circumstance is related to the mechanical nature of the setup. In abstracted physical terms, two springs are arranged in parallel (fixator and healing bone) and share the load at different distances to the center of force. This configuration leads to a steep decline of the fixator load already at small stiffness gain at the fracture. Already in 1972 Bourgois and Burny conducted mathematical modeling of the situation and reported an “hyperbolic pattern” of the loading curve [23]. Jenkins and Nokes confirmed this observation [21], which is also reflected in our RIL curves, particularly in the fast-healing animals, where the signal plateaus already between week 8 and 12 at a RIL of 10–20%. Subsequently, the residual signal remains comparatively stable and unaffected by late-stage healing processes, such as bone remodeling. This suggests that the main clinical advantage of the method lies in detecting healing onset early (respectively detecting non-healing early) because it is sensitive for initial fracture stiffness changes, whereas the ability of resolving the mechanical maturation during late healing phases (e.g., for determining the timepoint for implant removal) might be limited. Understanding the strengths and weaknesses of a method is crucial to define the suitable clinical scope and indication.

Research has moved then into proposing internal sensor systems, e.g., integrated into an intramedullary nail [24] or into a side-plate for proximal femur fracture fixation, battery powered [25] or passively powered [26]. Seide et al. instrumented a bone plate with a passive sensor, employed ground reaction force measurements and applied it to nonunion patients [8]. Kienast et al. [9] reported further clinical data from the same system and categorized patients into different healing patterns similar to Burny et al. [22]. New device proposals are constantly upcoming [27,28,29,30,31,32,33,34,35,36,37] underlining the importance of the topic.

These extensive academic efforts build the basis for our developments. There is undisputed value of evidence-based data collection for bone healing research, for optimizing implant designs or treatment strategies/therapies to advance trauma care [16,22,38]. However, there might also be strong potential for creating individual patient benefits by utilizing the principle in a clinical context. Use of implant load data for controlling patient mobilization could be one major clinical pillar. Implant load curve characteristics may be used to objectively steer the weight-bearing behavior of the patient [8,9,22,38]. Concretely, an apparent drop in the curve can be used to trigger increased weight-bearing. The benefit for the patient is anticipated as (1) earlier mobilization and hence earlier regain of life quality [8,9,38]; and (2) more confident mobilization due to decreased likelihood of implant failure through overloading [8,22]. For example, detection of loading spikes as seen in this study (Figure 8) may serve as an important indicator for identifying and preventing implant failures. However, it is unclear to what extent such measurements beyond the elastic range of the implant affect linearity of the sensor signal. Absolute magnitudes of loading spikes must hence be interpreted with care.

A second important pillar to create patient benefits is use of the sensor output to diagnose and react. Early identification of healing disturbances enables earlier intervention, which is finally thought to shorten the overall recovery time [8,16,38,39,40]. Simpson [40] went even further and stated that there may not only be a time benefit by treating a nonunion earlier (thereby benefiting from an earlier effect of the treatment), but also suggested that the success rate of treating a non-established nonunion may be higher than treating an established nonunion at a later stage. This hypothesis needs, however, clinical verification. In contrast to enabling early intervention, Kienast et al. [9] suggested that the benefit of implant load measurement may be the opportunity to avoid revision treatment in the first place when evidence is available that the healing is slowly but steadily progressing. Finally, reduction of X-ray exposure to patient and health care professionals is the apparent and obvious advantage of all radiation-free assessment methods.

In view of these significant clinical opportunities, development of the Fracture Monitor is the attempt to translate the groundwork of mentioned research groups into clinical practice and is therefore executed in a suitable regulatory environment following the requirements of the Medical Device Regulation (MDR). The hereby presented animal study provides the preclinical evidence of the system regarding safety and performance. The principle of continuous fracture monitoring was already proven in previous experiments using earlier designs and prototypes. In [11] we demonstrated the feasibility of the idea for the first time in vivo with a custom-made axially telescoping bone plate with integrated displacement sensor. The same research implant system was used in [41] to collect animal data stressing the importance of the early healing phase and day-one fracture stimulation. Designs of a bone plate attachable system were presented in [42] and [10]. Clinical data have been collected with a sensor version attachable to external fixator rods, proving the principle in a highly complex setting in terms of mechanical configuration (six-strut Taylor Spatial Frame) and biological conditions (infected tibia fractures with segment transport) [12]. Data from 10 patients show the general ability of the system to distinguish between healing and non-healing patients based on a 50% cut-off value of relative implant load drop.

This study has certain limitations. A control group without implanted datalogger to exclude potential adverse effects of the sensor presence on healing or on implant stability is missing. However, the animals showed potent callus formation and did not exhibit implant failures. Extending the study with additional control animals would, hence, be ethically questionable. Longitudinal measurements of ground reaction forces were omitted. Such refence data would, however, be valuable to improve interpretability of the system output and should be considered in future investigations. The simple CT-based healing metric (normalized bone volume) could only be evaluated in the lateral half of the defect due to metal artifacts close to the bone plate. To enhance the meaning of image-based measurements towards actual mechanical competence of the fracture, future studies may extend into finite elements modelling. The acquired mechanical test results of the healed bones represent another relevant metric for sensor output validation. However, after 9 months, all differences in healing were evened out, which disqualified this approach. We decided for a long observation period to investigate long-term sensor function and safety, but thereby scarified the ability to verify the device output with actual mechanical reference data. The study design targeted the creation of various gradations of healing as opposed to the conventional group-based approach (healing vs. nonhealing). The ability of the system to function as a healing/non-healing classifier was, hence, not tested in this study. Generally, the controlled environment of this animal experiment without clinical nonunion model using standardized osteotomies and fixation constructs does not permit direct translation of the results to the human situation. Thus, the next steps of the project are to finalize validation testing and to enter into a first-in-men clinical trial.

## 5. Conclusions

In this animal experiment, safety and performance of a continuously operating implant load sensor system was investigated. The implanted devices functioned reliably and uneventfully. The relative implant load measurements correlated significantly with the bone consolidation metrics, derived from radiographic images and CT scans. Continuous implant load monitoring appears, hence, relevant in supporting and objectifying fracture healing assessment and carries a strong potential to enable patient-tailored after-care in the future.

## Figures and Tables

**Table 1:** Study parameters and outcomes. SS = Stainless Steel, Ti = Titanium, R = right, L = left, RIL = relative implant load and LSB = least significant bits. * mRUST scoring not performed.

Animal ID	Side	Plate Material	Gap Width	Monitoring Period	Loading Events		Active Time	Time to 50% RIL	RIL End Level	Device Sensitivity (Plate Specific)
			mm	days	total	avg/day	avg %/day	days	%	LSB/kg
519030 *	R	SS	0.6	227	119,462	872	5.9	31	21	16.4
**519042**	L	Ti	0.6	252	194,460	1350	6.07	30	16	31.6
519031 *	L	SS	2	207	147,350	1002	7.4	23	11	14.6
**519043**	R	Ti	2	232	127,981	908	5.88	34	9	36.2
**519051**	R	SS	4	245	181,762	1387	7.29	24	10	15.9
**519049**	L	Ti	4	197	123,954	1158	5.32	22	11	24.7
**519050**	L	SS	6	250	271,940	1915	6.71	63	15	15.0
**519052**	R	Ti	6	245	146,161	981	7.37	45	3	27.2
519032 *	R	SS	10	209	205,236	1567	7.5	20	16	14.2
**519044**	L	SS	10	216	255,216	1701	6.62	82	11	18.6
**520001**	R	SS	30	255	213,563	1396	5.54	234	56	16.4
**mean**				230	180,644	1294	6.51	55	16	21.0
**SD**				21	52,730	343	0.81	62	14	7.7
**min**				197	119,462	872	5.32	20	3	14.2
**max**				255	271,940	1915	7.50	234	56	36.2

**Table 2:** Scoring scheme for the modified RUST [13]. Individual scores were cumulated for all three assessed cortices, resulting in a total score from 3 to 12.

Score Per Cortex	Callus	Fracture Line
1	Absent	Visible
2	Present	Visible
3	Bridging	Visible
4	Remodeled	Invisible

## Data Availability

Not applicable.

## Supplementary Materials

The following supporting video can be downloaded at https://www.mdpi.com/article/10.3390/medicina58070858/s1: Video S1: Exemplary livestream of the sensor signal recorded 4 weeks post-op for animal 519042.
